# Cornelia de Lange syndrome with *NIPBL* mutation and mosaic Turner syndrome in the same individual

**DOI:** 10.1186/1471-2350-13-43

**Published:** 2012-06-07

**Authors:** Jolanta Wierzba, María Concepción Gil-Rodríguez, Anna Polucha, Beatriz Puisac, María Arnedo, María Esperanza Teresa-Rodrigo, Dorota Winnicka, Fausto G Hegardt, Feliciano J Ramos, Janusz Limon, Juan Pié

**Affiliations:** 1 Department of Pediatrics, Hematology, Oncology and Endocrinology, Department of General Nursery, Medical University of Gdańsk, Gdańsk, Poland; 2 Unit of Clinical Genetics and Functional Genomics, Departments of Pharmacology-Physiology and Pediatrics, Medical School, University of Zaragoza, and Institute of Health Sciences of Aragón, Zaragoza, Spain; 3 Department of Pediatrics, Hematology and Oncology, Children University Hospital, Lublin, Poland; 4 Department of Biochemistry, School of Pharmacy, University of Barcelona, and Ciber-Obn, Health Institute Carlos III, Barcelona, Spain; 5 Department of Biology and Genetics, Medical University of Gdańsk, Gdańsk, Poland; 6 Unit of Clinical Genetics and Functional Genomics, Department of Pharmacology and Physiology, University of Zaragoza, Medical School, c/Domingo Miral s/n, Zaragoza, E-50009, Spain

**Keywords:** Cornelia de Lange syndrome, CdLS, *NIPBL*, Turner syndrome, TS, Monosomy X mosaicism, Mosaic 45,X/46,XX karyotype

## Abstract

**Background:**

Cornelia de Lange syndrome (CdLS) is a dominantly inherited disorder characterized by facial dysmorphism, growth and cognitive impairment, limb malformations and multiple organ involvement. Mutations in *NIPBL* gene account for about 60% of patients with CdLS. This gene encodes a key regulator of the Cohesin complex, which controls sister chromatid segregation during both mitosis and meiosis. Turner syndrome (TS) results from the partial or complete absence of one of the X chromosomes, usually associated with congenital lymphedema, short stature, and gonadal dysgenesis.

**Case presentation:**

Here we report a four-year-old female with CdLS due to a frameshift mutation in the *NIPBL* gene (c.1445_1448delGAGA), who also had a tissue-specific mosaic 45,X/46,XX karyotype. The patient showed a severe form of CdLS with craniofacial dysmorphism, pre- and post-natal growth delay, cardiovascular abnormalities, hirsutism and severe psychomotor retardation with behavioural problems. She also presented with minor clinical features consistent with TS, including peripheral lymphedema and webbed neck. The *NIPBL* mutation was present in the two tissues analysed from different embryonic origins (peripheral blood lymphocytes and oral mucosa epithelial cells). However, the percentage of cells with monosomy X was low and variable in tissues. These findings indicate that, ontogenically, the *NIPBL* mutation may have appeared before the mosaic monosomy X.

**Conclusions:**

The coexistence in several patients of these two rare disorders raises the issue of whether there is indeed a cause-effect association. The detailed clinical descriptions indicate predominant CdLS phenotype, although additional TS manifestations may appear in adolescence.

## Background

Cornelia de Lange syndrome (CdLS; OMIM 122470, 300590, and 610759) is an inherited congenital developmental disorder characterized by distinctive features including facial dysmorphism, growth and cognitive impairment, limb malformations, hirsutism and the involvement of other organ systems with variable expressivity [1]. Prevalence estimates range from 1:62,000 to 1:45,000 live births [2]. To date, mutations in three genes have been identified in ~65% of clinically well-defined CdLS cases, namely: *NIPBL* on chromosome 5p13 (60%), *SMC1A* on chromosome Xp11 and *SMC3* on chromosome 10q25 (5%) [3-6]. These three genes encode regulatory or structural components of the highly conserved Cohesin complex, which participates in chromosome segregation, DNA repair mechanisms, gene expression and chromosome conformation [7].

Turner syndrome (TS) is a common chromosomal disorder, usually associated with short stature, gonadal dysgenesis, cardiovascular abnormalities, hearing loss, neck webbing and lymphedema; although a number of organ systems and tissues may also be affected to a lesser or greater extent [8]. TS affects about one in 2000 live born females and results from complete or partial absence of one of the X chromosomes, frequently accompanied by cell-line mosaicism, which may also be tissue-specific [9,10].

Chromosomal rearrangements in individuals with CdLS have been reported over the years, involving 1–5, 7–14, 17, 18, 21 and X chromosomes [11]. To date, only four patients with CdLS have been reported to have sex chromosome anomalies: one male with 45,X/46,XY mosaicism [12], one female with 45,X karyotype [13] and two females with mosaic 45,X/46,XX karyotypes [14,15].

We report a female with CdLS, with an identified mutation in the *NIPBL* gene, and TS due to a mosaic 45,X/46,XX karyotype. We present a detailed phenotype description focusing on the typical clinical features of CdLS and TS. Furthermore, we compare the phenotype of our patient to other reported cases with similar karyotype and an unknown or different genotype. Finally, we examine the significance of a possible association of both syndromes.

## Case presentation

**Figure 1:**
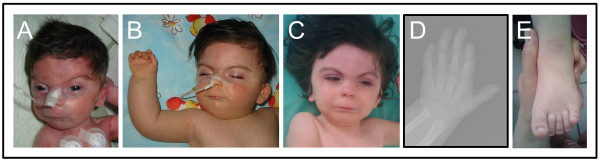
**Phenotype of the patient.** ( **A**) Frontal view of the patient in the first week of life. ( **B**) Frontal view and right hand of the patient at the age of 2 years and 10 months; and ( **C**) at 3 years. ( **D**) Roentgenogram of the left hand at the age of 3 years and 6 months and ( **E**) left foot of the patient at the age of 3 years. Note typical CdLS facial features seen in our patient included synophrys, arched eyebrows, long eyelashes, depressed nasal bridge and anteverted nares, long and flat philtrum, thin upper lip, downslanting corners of the mouth, low set and posteriorly rotated ears and microbrachycephaly ( **A**- **C**). Several mild musculoskeletal anomalies were noted including small hands and feet ( **D** and **E**), clinodactyly of the fifth finger and proximally placed thumb ( **D**).

The patient is the first child of a healthy and unrelated 35-year-old father and a 37-year-old mother. There was no family history of congenital defects. She has a healthy younger brother. The girl was born at 35 weeks gestation by caesarean section due to placental insufficiency. Birth weight was 1.350 kg, length 43 cm and head circumference 25 cm (all below the 3^rd^ centile for gestational age) (Table 1). Apgar score was 7 in the first minute and 9 at five minutes. Craniofacial dysmorphism included: microbrachycephaly, bitemporal narrowing distance, synophrys, arched eyebrows, long and irregularly placed eyelashes, depressed nasal bridge, anteverted nares, long and flat philtrum, thin upper lip, downslanting corners of the mouth, micrognathia, high arched and vaulted palate, low-set and posteriorly rotated ears, low posterior hairline, short and webbed neck and hirsutism (Figure 1A and B, Table 1). She had small hands and feet, lymphedema of the feet (resolved at two months of age), bilateral clinodactyly of the fifth finger, pro-ximally placed thumbs, single palmar crease and hip dislocation (Figure 1B, D and E, Table 1). Additional neonatal findings included mild hypertonia, lack of the sucking reflex, congenital bilateral glaucoma, retinopathy, atrial and ventricular septal defect (ASD - VSD) and mild pulmonary stenosis (PS) that did not require surgery. At two years of age gastroesophageal reflux disease (GERD) was suspected although it could not be confirmed. More detailed clinical description of the patient is provided in Table 1.

**Table 1:** Clinical data for patients with typical features of CdLS and TS

**Clinical findings**		**CdLS and 45,X/46,XX mosaicism**[14]		**CdLS (*NIPBL*mutation) and 45,X/46,XX mosaicism [this paper]**		**CdLS (*SMC1A*mutation) and 45,X/46,XX mosaicism**[15]	
		***TS***	***CdLS***	***TS***	***CdLS***	***TS***	***CdLS***
***Birth parameters and growth***							
** Birth weight** < 10^th^ centile		-	+	-	+	-	-
** Length at birth** < 10^th^ centile		-	+	-	+	N/A	N/A
** Growth:** Short stature		+	+	+	+	+	+
***Craniofacial features***							
**Eye**	Synophrys	-	+	-	+	-	+
	Arched eyebrows	-	+	-	+	-	+
	Long eyelashes	-	+	-	+	-	-
**Nose**	Depressed nasal bridge	-	+	-	+	-	-
	Anteverted nares	-	+	-	+	-	-
**Philtrum**	Long	-	+	-	+	-	+
	Prominent	-	+	-	+	-	-
	Smooth	-	+	-	+	-	+
**Mouth**	Thin upper lip	-	+	-	+	-	+
	Down-slanting corners	-	+	-	+	-	-
	Widely-spaced teeth	-	+	-	-	-	+
	Micrognathia	+	+	+	+	+	+
**Ear**	Low-set	+	+	+	+	-	-
	Posteriorly rotated	-	+	-	+	-	-
**Neck**	Low posterior hairline	+	+	+	+	-	-
	Short neck	+	+	+	+	+	+
	Webbed neck	+	-	+	-	-	-
**Skull**	Microbrachycephaly	-	+	-	+	-	+
	Bitemporal narrowing distance	-	+	-	+	-	+
	Skull asymmetry with right-sided flattening	-	-	-	-	-	+
***Neurology and cognitive profile***							
** Neurological involvement:** Hypertonia		-	+	-	+	N/A	N/A
** Cognitive: Mental retardation**		N/A	N/A	-	+	-	+
** Verbal and motor developmental delay**							
Speech delay		+	+	+	+	+	+
Language delay		-	+	-	+	-	+
Developmental delay		-	+	-	+	-	+
***Musculoskeletal system***							
Small hands and/or feet		-	+	-	+	-	+
5^th^ finger clinodactyly		-	+	-	+	N/A	N/A
Syndactyly 3^rd^-4^th^ fingers		-	-	-	-	-	+
Proximally placed thumb		-	+	-	+	-	+
Single palmar crease		-	-	-	+	N/A	N/A
Wide Space 1^st^ 2^nd^ toes		-	-	-	-	-	+
Bilateral cubitus valgus		-	-	+	-	N/A	N/A
Limited elbow extension		-	+	-	+	N/A	N/A
Hip dislocation		-	-	+	+	N/A	N/A
Short sternum		-	-	+	-	N/A	N/A
Scoliosis		-	-	-	-	+	+
Bone age retardation		-	-	+	-	N/A	N/A
Broad chest with widely-spaced nipples		+	-	+	-	+	-
***Ophthalmologic findings***							
Glaucoma		-	-	+	+	N/A	N/A
Retinopathy		N/A	N/A	-	+	N/A	N/A
Myopia		N/A	N/A	+	+	N/A	N/A
***ENT manifestations***							
Sensorineural hearing loss		N/A	N/A	+	+	N/A	N/A
***Skin and nails***							
Lymphedema of the feet		-	-	+	-	-	-
Hirsutism		-	+	-	+	-	-
Cutis marmorata		-	-	-	+	N/A	N/A
Small and hypoplastic nails		+	-	+	-	-	-
Hyperconvexed nails		-	-	-	-	+	-
***Cardiac system***							
ASD, VSD and PS		-	-	-	+	-	-
Tetralogy of Fallot		-	-	-	-	-	+
***Gastrointestinal system***							
GERD		+	+	+	+	+	+
Gastrointestinal malrotation		-	-	-	-	-	+
Constipation		-	-	-	+	-	+
Feeding problems		-	-	+	+	+	+
Poor sucking and swallowing reflexes		+	-	+	-	N/A	N/A

(+) Present; (−) Not present; N/A: Not available; OFC: Occipito-Frontal Circumference; ENT: Ear-Nose-Throat; ASV: Atrial Septal Defect; VSD: Ventricular Septal Defect; PS: Pulmonary Stenosis; GERD: Gastroesophageal Reflux Disease.

At the age of 3 years and 6 months (Figure 1C-E) her weight was 9.1 kg, height 81 cm and head circumference 41 cm (≤ 50^th^ centile on CdLS growth charts).Physical examination showed broad chest with widely spaced nipples, short sternum, bilateral cubitus valgus, limited elbow extension, small and hypoplastic nails and myopia. Developmental milestones were severely delayed. She was able to sit unsupported, but not to stand or walk. Speech was absent but she could follow simple instructions. The patient had autistic-like features with episodes of aggression and self-injurious behaviour. Mild bilateral sensorineural hearing loss was detected by auditory brainstem response (ABR). She had delayed bone age (Figure 1D) (Table 1). Biochemical, endocrine and metabolic studies were normal, except for high serum TG (triglyceride) levels (232 mg/dL; normal value range for TG levels is < 98 mg/dL). Thyroid function tests (T3, T4, TSH) and celiac screen (IgA-TTG and IgA-EmA antibodies) were also normal.

## Methods and results

### Molecular analysis

Blood samples and buccal smears were obtained after written informed consent, and genomic DNA was isolated from peripheral blood lymphocytes and oral mucosa epithelial cells by standard protocols. The entire coding region and flanking intron sequences of *NIPBL* (exons 2–47) were screened for mutations by bidirectional sequencing. The *NIPBL* reference sequence used was NM_133433. Parental genotypes were screened to assess whether the variant was *de novo* or inherited.

**Figure 2:**
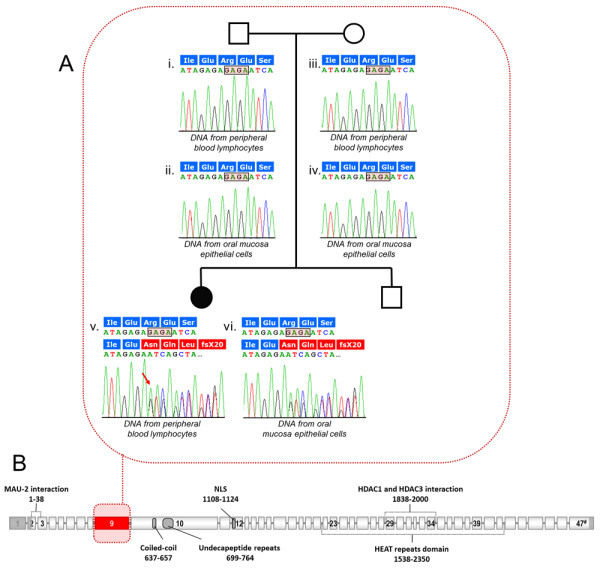
**(A) Pedigree of the affected family and partial electropherograms of exon 9 of the* NIPBL *gene.** White symbols indicate unaffected individuals, whereas black symbol indicate the affected individual. The sequencing analysis performed on genomic DNA from the patient peripheral blood lymphocytes (v) and oral mucosa epithelial cells (vi) shows similar heights of the peaks of the allele carrying the c.1445_1448delGAGA mutation in both tissues. Wild-type electropherograms identified in the normal parents are also indicated (i-iv). **(B)** Schematic model of the *NIPBL* gene. Sequence features of human NIPBL protein previously reported are indicated [23,30] . The open reading frame of *NIPBL* gene is marked in light grey. The exon 9 of the *NIPBL* gene is highlighted in red.

*NIPBL* mutational screening showed a *de novo* mutation in exon 9 (c.1445_1448delGAGA), which predicts a truncated protein p.R482NfsX20 (Figures 2A and B). To test whether the patient carries the *NIPBL* mutation in mosaic state, molecular analyses were performed on two tissues of different embryonic origins: peripheral blood lymphocytes (mesoderm) and epithelial cells from oral mucosa (ectoderm). The mutation-related peaks were similar in both tissues, ruling out widespread mosaicism (Figure 2A).

### Cytogenetic analysis

**Figure 3:**
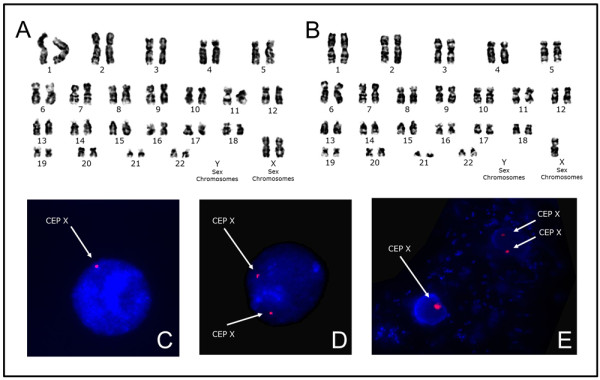
**GTG-banded and FISH images from lymphocytes and buccal epithelial cells.** Panels ( **A**- **B**) show karyotypes of peripheral blood lymphocytes demonstrating the 45,X/46,XX mosaicism. Panels ( **C**- **E**) show FISH analysis using CEP X Spectrum Orange/Y Spectrum Green Direct Labelled Fluorescent DNA probes from Abbott Molecular within of interphase nuclei of lymphocytes ( **C**- **D**) and buccal epithelial cells ( **E**). ( **C**) and ( **E**) show FISH interphase cells with one copy of the X chromosome (arrow). ( **D**) and ( **E**) show the presence of two copies of the X chromosomes (double arrows). Only representative cells with different karyotypes are shown here.

Conventional cytogenetic analysis of metaphase chromosomes prepared from cultured peripheral blood lymphocytes was performed according to standard procedures using the GTG banding technique. Karyotype was 45,X/46,XX, with 24% cells containing only one X chromosome (Figures 3A and B). The parents’ karyotypes were also examined and both were normal.

### Fluorescence *in situ* hybridization (FISH) analysis

FISH analyses were performed on cultured peripheral blood lymphocytes and oral mucosa epithelial cells of the patient. Interphase nuclei were hybridized with commercial CEP X Spectrum Orange/Y Spectrum Green Direct Labeled Fluorescent DNA Probes (Abbott Molecular) according to the manufacturer’s instructions. For each analysis, a minimum of 500 or 100 interphase nuclei from blood lymphocytes or oral mucosa epithelial cells were scored, respectively. FISH analyses confirmed the presence of 45,X/46,XX mosaicism in both tissues, with 28% and 7% of monosomy X in peripheral blood lymphocytes and buccal smears, respectively (Figures 3C-E).

## Discussion

Here we report a patient with CdLS and a *NIPBL* frameshift mutation (c.1445_1448delGAGA deletion, p.Arg482AsnfsX20), who also had mosaic TS. Clinical diagnosis of CdLS was suspected from the typical craniofacial features, hirsutism, pre- and post-natal growth retardation, congenital heart defects and delayed psychomotor development with specific behavioural problems (Table 1). Following the scoring system for severity proposed by Kline et al. [2007] [16], she has a severe CdLS phenotype despite the mild anomalies of the upper limbs. In fact, the same *NIPBL* mutation was previously identified in another female with CdLS from Portugal, who had a similar phenotype [17]. Interestingly, our patient also showed peripheral lymphedema and webbed neck in the neonatal period [10] (Table 1), suggesting the diagnosis of TS, which was subsequently confirmed by cytogenetic analysis.

Ophthalmologic findings have been reported in a high percentage of CdLS and TS patients. However, the congenital bilateral glaucoma diagnosed in our case has been described in only three patients with CdLS, and in three other patients with mosaic TS [18-20].

Congenital heart defects (CHD) are common both in TS (17-45%) [10,21] and in CdLS (13-70%) [16,22]. Our case has CHD that are common in CdLS (ASD, VSD and PS) but rarely seen in TS (<0.5%) [16,21-23].

To date, only four patients with chromosomal rearrangements involving sex chromosomes have been reported in CdLS [12-15]. Only two of those cases had detailed clinical description and mosaic TS karyotype and could be compared to our patient [14,15] (Table 1).

The genotype of the first case, reported by Klosovskiĭ et al. in 1968, is still unknown [14]. Like our patient she was diagnosed during childhood, and she also showed similar TS features and severe CdLS phenotype (Table 1).

More recently, Hoppman-Chaney et al. reported a female patient with a novel multi-exon deletion of the *SMC1A* gene, who showed an unusual, severe phenotype of CdLS and a mosaic monosomy X (35% of peripheral blood lymphocytes) [15] (Table 1). Remarkably, her clinical findings related to mosaic TS were fewer and milder than in our patient. She had broad chest with wide-set nipples and hyperconvexed fingernails [15] (Table 1). This could be explained by the highly variable phenotypic expression of mosaic TS individuals [24]. Moreover, she also presented with atypical facial features for CdLS, such as prominent metopic suture, sparse hair, deep-set eyes and long and narrow earlobes [15]. She also showed severe typical features of classic CdLS, rarely seen in affected females with *SMC1A* mutations [3,15,23,25] (Table 1). This discrepancy could be due to the nature of her mutation, which causes severe protein dysfunction, similar to that caused by truncating mutations in the *NIPBL* gene [3,15,23,25,26].

Molecular genetic analysis in our patient identified a *de novo* heterozygous frameshift mutation in exon 9 of the *NIPBL* gene (c.1445_1448delGAGA), resulting in a predicted stop codon and truncation of the translational product (p.R482NfsX20). Six additional truncating mutations have been found inside exon 9 [17,27], which is the second longest coding exon in *NIPBL* gene (627 base pairs). They are located within the N-terminal half of the protein, which is apparently only conserved in vertebrates and where most of the truncating mutations have been identified (~70% *vs* 42% C-terminal half). These data suggest this domain is important, although it has not yet been associated with any specific function [23].

FISH analyses of tissues from different germ layers revealed a low level of mosaicism for monosomy X. However, the *NIPBL* mutation was identified in all the tissues analyzed, ruling out somatic mosaicism. These findings suggest that, ontogenically, the *NIPBL* mutation appeared earlier than the aneuploidy for the X chromosome.

Surprisingly, frameshift mutations in exon 9 of *NIPBL* have also been identified in some gastrointestinal cancers associated with chromosomal instability and aneuploidy [7,28,29]. It has been proposed that these mutations could alter chromosome segregation, the canonical role for the Cohesin complex, leading to chromosome imbalance with chromosome loss or gain [25,28]. This hypothesis may provide an explanation for the aneuploidy in our case, since a common cause of mosaicism is nondisjunction in an early postzygotic mitotic division. Hence, we suggest that the mutation in NIPBL could be the cause of the monosomy of the X chromosome. Moreover, this hypothesis would explain the numerous reports of individuals clinically diagnosed with CdLS who also carried a chromosomal abnormality [11,15]. Further experiments will be needed to confirm this association.

## Conclusions

Here, we report a patient with CdLS due to a mutation in the *NIPBL* gene and mosaic TS. This patient showed the classical phenotype of CdLS, although without limb reduction. She was also clinically diagnosed with TS because of two typical recognizable features of the syndrome: the peripheral lymphedema and the webbed neck. Molecular characterization showed that the *NIPBL* mutation was present in all the tissues analyzed from different embryonic origins (mesoderm and ectoderm), while FISH analyses revealed that the mosaicism for the monosomy of the X chromosome was tissue specific. These findings indicate that, ontogenically, the *NIPBL* mutation appeared before the monosomy X. Moreover, the recent identification of frameshift mutations in exon 9 of the *NIPBL* gene in colon cancer cells associated with chromosome aneuploidy suggests that the NIPBL mutation could contribute to the loss of the X chromosome [28].

## Consent

The manuscript was written with the approval of Independent Bioethics Committee for Clinical Research, Medical University of Gdańsk. Written informed consent was obtained from the patient’s parents for publication of this case report and any accompanying images. A copy of the written consent is available for review by the Series Editor of this journal.

## List of abbreviations

CdLS, Cornelia de Lange syndrome; TS, Turner syndrome; OFC, Occipito-Frontal Circumference; ENT, Ear-Nose-Throat; ASV, Atrial Septal Defect; VSD, Ventricular Septal Defect; PS, Pulmonary Stenosis; GERD, Gastroesophageal Reflux Disease; ABR, Auditory Brainstem Response; IgA-TTG, IgA anti-tissue transglutaminase antibody; IgA-EmA, IgA anti-endomysial antibody; CHD, Congenital Heart Defect.

## Competing interests

The authors declare that they have no competing interests.

## Authors’ contributions

JW, JL, FGH and JP participated in the conception and the design of the study. MCGR and BP designed and interpreted the molecular evaluations. MCGR, MA and METR conducted the molecular analyses. DW performed cytogenetic and FISH analyses. JW, AP and FJR examined the patient, collected the data relevant to this case report and made the clinical diagnosis of the patient. JW, MCGR and JP reviewed the literature and wrote the MS. All the authors have read, revised and approved the final version of the manuscript.

## Pre-publication history

The pre-publication history for this paper can be accessed here:

http://www.biomedcentral.com/1471-2350/13/43/prepub
